# Impact of frailty on long-term mortality in older patients receiving intensive care via the emergency department

**DOI:** 10.1038/s41598-023-32519-2

**Published:** 2023-04-03

**Authors:** Mototaka Inaba, Hiromichi Naito, Takashi Yorifuji, Chikaaki Nakamichi, Hiroki Maeyama, Hideki Ishikawa, Nobuaki Shime, Sadayori Uemori, Satoshi Ishihara, Makoto Takaoka, Tsuyoshi Ohtsuka, Masahiro Harada, Satoshi Nozaki, Keisuke Kohama, Ryota Sakurai, Shuho Sato, Shun Muramatsu, Kazunori Yamashita, Toshihiko Mayumi, Kaoruko Aita, Atsunori Nakao, the LIFE Study Investigators, Satoshi Mochizuki, Hirofumi Itoh, Asase Senda, Kana Otani, Chison Gon, Shunsuke Taito, Takeshi Ohnishi, Yuji Taguchi, Toru Miike, Koki Umeda, Yuji Kondo, Takao Arai, Junya Tsurukiri, Kaoru Masuda

**Affiliations:** 1 Department of Emergency, Critical Care, and Disaster Medicine, Okayama University Graduate School of Medicine, Dentistry, and Pharmaceutical Sciences, 2-5-1 Shikata, Okayama, 700-8558 Japan; 2 Department of Epidemiology, Okayama University Graduate School of Medicine, Dentistry, and Pharmaceutical Sciences, Okayama, Japan; 3 Advanced Emergency and Critical Care Center, National Hospital Organization, Nagasaki Medical Center, Omura, Japan; 4 Emergency and Critical Care Center, Tsuyama Chuo Hospital, Tsuyama, Japan; 5 Trauma and Resuscitation Center, Teikyo University Hospital, Tokyo, Japan; 6 Department of Emergency and Critical Care Medicine, Graduate School of Biomedical and Health Sciences, Hiroshima University, Hiroshima, Japan; 7 Department of Emergency, Yodogawa Christian Hospital, Osaka, Japan; 8 Department of Emergency and Critical Care, Hyogo Emergency Medical Center, Kobe, Japan; 9 Acute Care Division, Harima-Himeji General Medical Center, Himeji, Japan; 10 Emergency Department, National Hospital Organization Yokohama Medical Center, Yokohama, Japan; 11 Department of Emergency and Critical Care, National Hospital Organization Kumamoto Medical Center, Kumamoto, Japan; 12 Emergency Department, Okayama Saiseikai General Hospital, Okayama, Japan; 13 Department of Emergency, Disaster, and Critical Care Medicine, Hyogo College of Medicine, Nishinomiya, Japan; 14 Department of Emergency and Critical Care Medicine, Faculty of Medicine, Saga University, Saga, Japan; 15 Emergency Medical Center, Saiseikai Senri Hospital, Suita, Japan; 16 Emergency Department, Japanese Red Cross Medical Center, Tokyo, Japan; 17 Acute and Critical Care Center, Nagasaki University Hospital, Nagasaki, Japan; 18 Department of Emergency Medicine, University of Occupational and Environmental Health Hospital, Kitakyushu, Japan; 19 Uehiro Division, Center for Death and Life Studies and Practical Ethics, Graduate School of Humanities and Sociology, University of Tokyo, Tokyo, Japan; 20 Saiseikai Senri Hospital, Suita, Japan; 21 Yodogawa Christian Hospital, Osaka, Japan; 22 National Hospital Organization Nagasaki Medical Center, Omura, Japan; 23 Hiroshima University Hospital, Hiroshima, Japan; 24 Hyogo Emergency Medical Center, Kobe, Japan; 25 Steel Memorial Hirohata Hospital, Himeji, Japan; 26 Saga University Hospital, Saga, Japan; 27 University of Occupational and Environmental Health Hospital, Kitakyushu, Japan; 28 Japanese Red Cross Medical Center, Tokyo, Japan; 29 Tokyo Medical University Hachioji Medical Center, Hachioji, Japan

**Keywords:** Diseases, Health care, Medical research

## Abstract

The aim of this study was to evaluate whether frailty was associated with 6-month mortality in older adults who were admitted to the intensive care unit (ICU) with an illness requiring emergency care. The investigation was a prospective, multi-center, observational study conducted among the ICUs of 17 participating hospitals. Patients ≥ 65 years of age who were admitted to the ICU directly from an emergency department visit were assessed to determine their baseline Clinical Frailty Scale (CFS) scores before the illness and were surveyed 6 months after admission. Among 650 patients included in the study, the median age was 79 years old, and overall mortality at 6 months was as low as 21%, ranging from 6.2% in patients with CFS 1 to 42.9% in patients with CFS ≥ 7. When adjusted for potential confounders, CFS score was an independent prognostic factor for mortality (one-point increase in CFS, adjusted risk ratio with 95% confidence interval 1.19 [1.09–1.30]). Quality of life 6 months after admission worsened as baseline CFS score increased. However, there was no association between total hospitalization cost and baseline CFS. CFS is an important predictor of long-term outcomes among critically ill older patients requiring emergent admission.

## Introduction

Indications for intensive care in vulnerable older adults are often a significant concern for physicians in the emergency department^1^. The decision to admit a patient to the intensive care unit (ICU) must take the patient's age, underlying medical conditions, and age-related vulnerabilities such as frailty into account^2^. Frailty is defined as a clinically recognized state of increased vulnerability in older adults^3^. To assess frailty, several scales commonly derived from two major definitions have been used^4,5^. The concept of frailty is widely accepted in geriatric medicine. Frailty is a clinical syndrome characterized by increased vulnerability to stressors leading to functional impairment and adverse health outcomes. Frailty is associated with complications, disability, and mortality, particularly after surgery^6,7^. With global increases in the aging population and in consideration of their physiological and cognitive vulnerabilities, there has been a focus on the impact of frailty on treatment outcomes in emergency and critical care medicine^8,9^. However, evidence to guide the treatment and management of older patients in the ICU is still limited. Moreover, there are currently no international recommendations detailing the indications for treatment or admission to the ICU for critically ill older patients. Furthermore, information on the impact of frailty in the emergency and intensive care settings on long-term outcomes is limited.

In this prospective study, we aimed to evaluate the association between baseline frailty and 6-month mortality after ICU admission. The Clinical Frailty Scale (CFS) was used to evaluate patients 65 years of age or older who were admitted to the ICU with an illness requiring emergency care. Six-month mortality and quality of life (QOL) were examined for each CFS score after adjustment for covariates.

## Methods

### Study design and setting

This prospective, observational study was conducted at medical facilities in Japan. Nationally certified emergency and critical care centers and Japanese Society of Intensive Care Medicine board-certified training facilities for intensive care specialists could participate in the study. The study protocol was approved by a suitable ethics committee at each institution and conforms to the provisions of the Declaration of Helsinki. Names of each ethics committee are listed in the acknowledgement section. Informed consent was obtained from each patient or their surrogate. The study was registered in the University Hospital Medical Information Network Clinical Trials Registry: UMIN-CTR (ID: UMIN000037430) and received funding from the Japan Society for the Promotion of Science (KAKENHI funds, Grants-in-Aid for Scientific Research).

### Patient enrollment

Patients were enrolled at 17 participating centers (Supplementary eTable 1 online) during four consecutive months at each facility from November 2019 through April 2020. Inclusion criteria were admission to the ICU directly from an emergency department visit (including inter-facility transfers) and age 65 years or older at the time of admission to the ICU. The decision to admit the patient to the ICU was left to the emergency physicians at each facility. Eligible patients were screened consecutively by the attending physicians or nurses at the time of ICU admission and were excluded if it was not possible to determine their baseline CFS score or obtain consent from patient/surrogate.

### Study sample size

Based on previous results^10^, mortality in the non-frail group was estimated as 30% and mortality in the frail group was estimated as 50%; α error was set at 0.05 with power set at 0.8. The required number of cases was calculated to be at least 206. Because a questionnaire was being administered to assess outcomes including mortality 6 months after admission, we estimated that the questionnaire return rate would be 60%. To ensure a sufficient number of cases for the planned, stratified analysis (three times more than the minimum required cases), we initially set the enrollment period at each location at 3 months with the goal to screen 1000 patients. We estimated that the questionnaire would be returned for 600 of these patients at the 6-month survey. One month after the start of enrollment, the number of enrolled patients was lower than expected, and the enrollment period was extended to 4 months.

### Data collection

#### Baseline patient characteristics

At the time of ICU admission, basic patient characteristics, such as age, sex, height, weight, Charlson Comorbidity Index (CCI) score, illness etiology, and illness severity were collected by the attending medical providers. Similarly, the patients' living conditions, education levels, and occupations before admission were collected through a written questionnaire completed at the time of ICU admission. Each patient’s clinical severity was recorded after admission using all information available, including Acute Physiology and Chronic Health Evaluation (APACHE) II score assessed in the ICU and treatment status, including the use of mechanical ventilation. Patient outcome data collected at the time of discharge included discharge destination, ICU and hospital length of stay, and total medical costs. Costs were converted to United States dollars (USD) using an exchange rate of 100 Japanese yen to 1 USD.

#### Clinical frailty scale

The CFS was proposed by Rockwood and colleagues as an index of frailty to classify the vulnerability of older adults into nine levels from 1 (very fit) to 9 (terminally ill)^7,11^. Higher scores indicate more frailty. We used a validated, Japanese-translated version of the CFS^12^. Prior to the study, standardized scoring sheets were distributed to each facility; then, the physicians and nurses were trained to obtain a baseline CFS score before the acute illness/injury (approximately 2 weeks) via interview with the patient/surrogate. A baseline CFS score was obtained for each patient immediately at ICU admission by the attending physician or nurse.

#### Follow-up patient data

Six months after admission, a survey sheet was mailed to the patient or their surrogate to determine mortality and QOL. When participants did not respond to the mailed survey, the research collaborator at each institution attempted to conduct a telephone survey or medical record survey to assess mortality within 6 months of admission. QOL was assessed using a validated Japanese version of the EQ-5D-5L questionnaire^13,14^. Participants were asked to complete the self-complete version when the individual was able to answer and the proxy version when a surrogate answered. EQ-5D-5L index was evaluated using the EQ-5D-5L Crosswalk Index Value Calculator (Japanese version). All data were entered into the Research Electronic Data Capture (REDCap) system, a web-based application^15^.

### Outcome measures

The primary outcome of the study was 6-month mortality. The secondary outcomes were 28-day mortality, QOL 6 months after ICU admission, discharge destination, and total hospitalization cost.

### Statistical analysis

Continuous variables are presented as median and interquartile range (IQR). Categorical variables are presented as numbers and percentages. Mortality was described for each CFS score. Due to the small sample size of patients classified as CFS 8 and no patients classified as CFS 9, CFS ≥ 7 were combined into one category for analysis.

We conducted Poisson regression with robust error variance to examine the association between CFS and mortality and obtained estimated risk ratios (RRs) with 95% confidence intervals (CIs) using a CFS score of 1 as a reference. We also estimated the RR per one unit increase in CFS. We first estimated crude RRs and then estimated adjusted RRs controlling for age, sex, CCI score, and APACHE II score. We also conducted stratified analysis separated by age, use of mechanical ventilation, exacerbation of pre-existing chronic illness, and illness severity. We compared EQ-5D-5L scores and total cost during hospitalization as secondary outcomes between CFS categories.

Because some patients were discharged within 24 h of ICU admission, the data necessary to evaluate APACHE II score could not be obtained for all participants. Of the 650 patients included in this study, 146 (22.5%) were missing APACHE II score data. Because missing values were likely to be lost at random and to avoid loss in statistical efficiency, missing APACHE II values were imputed using multivariate normal regression with multiple imputation (20 imputations). The primary analyses estimating RRs were based on imputed data, but we also conducted sensitivity analyses that included patients with complete data only. All statistical analyses were performed using STATA version 17 (StataCorp LP, College Station, TX).

### Ethical approval

The study protocol was approved by: Okayama University Graduate School of Medicine, Dentistry and Pharmaceutical Sciences and Okayama University Hospital, Ethics Committee, Yokohama Medical Center Independent Ethics Committee, Saiseikai Senri Hospital, Ethics Committee, Ethics Committee of Yodogawa Christian Hospital, Ngasaki Medical Center, Ethics Review Board, Okayamasaiseikai, Ethics Review Committee, Ethics Committee of Tsuyama Chuo Hospital, Teikyo University, Ethics Committee, Nagasaki University Hospital Clinical Research Ethics Committee, The Ethics Review Board of Hyogo College of Medicine, Hiroshima University Institutional Review Board, HEMC Ethics Committee, National Hospital Organization Kumamoto Medical Center, Ethics Committee, Steel Memorial HIROHATA Hospital Ethics Committee, Saga University Clinical Research Review Board, University of Occupational and Environmental Health, Ethics Review Committee, Japanese Red Cross Medical Center, Clinical Research Ethics Committee.

## Results

### Participants

**Figure 1:**
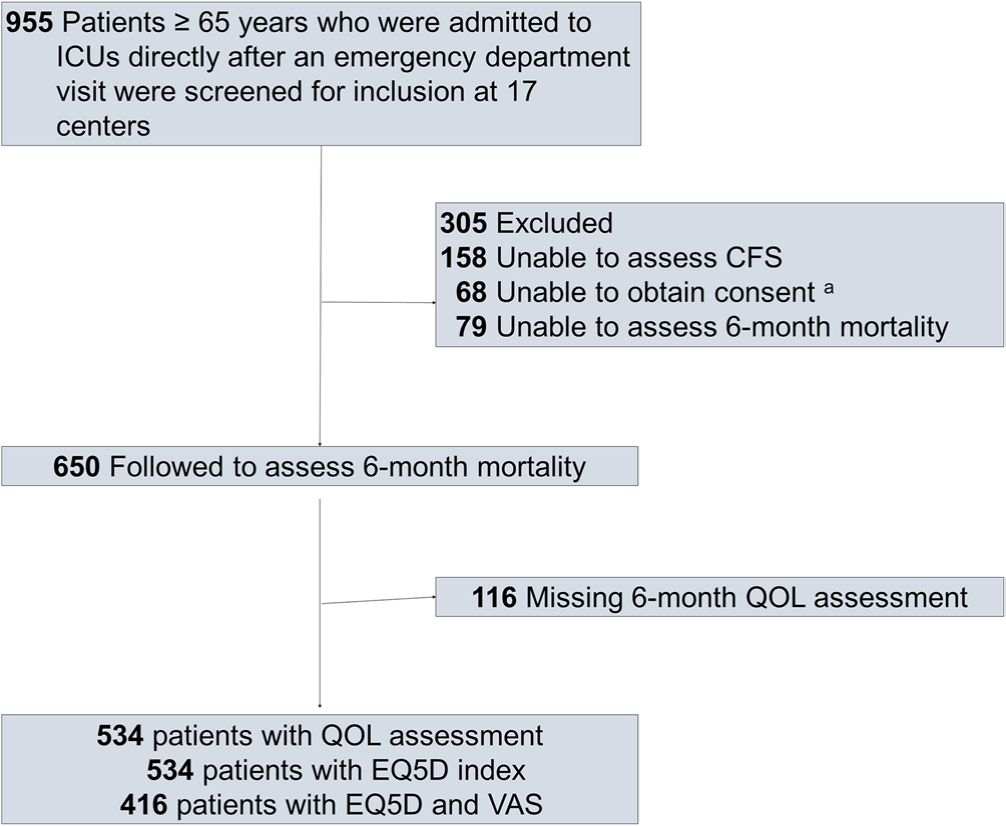
Flowchart detailing study participants. ^a^Consent was withdrawn for two patients. *ICU* intensive care unit, *CFS* Clinical Frailty Scale, *QOL* Quality of Life, *EQ-5D* EuroQol 5 Dimensions, *VAS* Visual Analog Scale.

In total, 955 patients were screened for eligibility, and 650 were included in the study (Fig. 1). QOL data were available in 534 patients. Baseline characteristics of the participants are described in Table 1. The participants’ median age was 79 years old (IQR 72–85); 58.5% were male. Most (84.3%) lived at home without assistance before admission; 6.8% were living at home with assistance. The remaining patients were admitted from a nursing home (6.5%) or hospital (2.5%). The median APACHE II score for these older patients was 22 (IQR 16–29), median CCI score was 5 (IQR 4–6), and median CFS was 3 (IQR 3–5). Frailty, as indicated by CFS ≥ 5, was observed in 173 patients (26.6%).

**Table 1:** Baseline patient characteristics.

Characteristic, n (%) unless otherwise stated	Total (n = 650)	Survivor (n = 514)	Death within 6 months (n = 136)
Age, median (IQR), years	79 (72–85)	78 (71–84)	83 (76–87)
Sex			
Male	380 (58.5)	293 (57.0)	87 (64.0)
Female	270 (41.5)	221 (43.0)	49 (36.0)
CCI score, median (IQR)	5 (4–6)	4 (3–6)	5 (4–6)
APACHE II score, median (IQR)ª	22 (16–29)	20 (15–26)	28 (22–35.5)
ICU admission category^b^			
Cardiology	145 (22.3)	121 (23.5)	24 (17.6)
Pulmonary	71 (10.9)	47 (9.1)	24 (17.6)
Gastrointestinal	93 (14.3)	76 (14.8)	17 (12.5)
Neurology	151 (23.2)	124 (24.1)	27 (19.9)
Trauma	96 (14.8)	78 (15.2)	18 (13.2)
Endocrine	33 (5.1)	23 (4.5)	10 (7.4)
Skin/tissue	7 (1.1)	6 (1.2)	1 (0.7)
Urology	7 (1.1)	5 (1.0)	2 (1.5)
Others	47 (7.2)	34 (6.6)	13 (9.6)
Daily living condition before emergent admission^c^			
Home without assistance	548 (84.3)	451 (87.7)	97 (71.3)
Home with assistance	44 (6.8)	31 (6.0)	13 (9.6)
Nursing home	42 (6.5)	28 (5.4)	14 (10.3)
Hospital	16 (2.5)	4 (0.8)	12 (8.8)
CFS score^d^			
1	81 (12.5)	76 (14.8)	5 (3.7)
2	66 (10.2)	57 (11.1)	9 (6.6)
3	187 (28.8)	166 (32.3)	21 (15.4)
4	143 (22.0)	104 (20.2)	39 (28.7)
5	58 (8.9)	40 (7.8)	18 (13.2)
6	59 (9.1)	39 (7.6)	20 (14.7)
7	43 (6.6)	28 (5.4)	15 (11.0)
8	13 (2.0)	4 (0.8)	9 (6.6)

*IQR* interquartile range, *APACHE II* Acute Physiology and Chronic Health Evaluation II, *CCI* Charlson Comorbidity Index, *CFS* Clinical Frailty Scale, *ICU* intensive care unit.

^a^Evaluated in 504 patients.

^b^Nine categories of illness etiology were defined by the research group. One category was selected by the attending physician for each patient.

^c^Four discharge status categories were defined by the research group and presented to the patient or surrogate, who then selected the most applicable category.

^d^No patient was scored as CFS 9.

Six-month surveys were conducted by mail, telephone, or for some patients, through review of outpatient medical records, but 79 patients (10.8%) or their surrogates did not respond. When we compared the characteristics of the 79 non-responders with the 650 responders, a significantly higher incidence of dementia (26% vs. 38.0%) or acute exacerbation of chronic diseases (13.8% vs. 29.1%) was observed in the nonresponding patients. However, clinical severity and CFS scores were not significantly different between responders and non-responders (Supplementary eTable 2 online). Treatments, treatment limitations, and other clinical data during hospitalization are shown in Supplementary eTable 3 and eTable 4 online.

### Primary outcome

**Figure 2:**
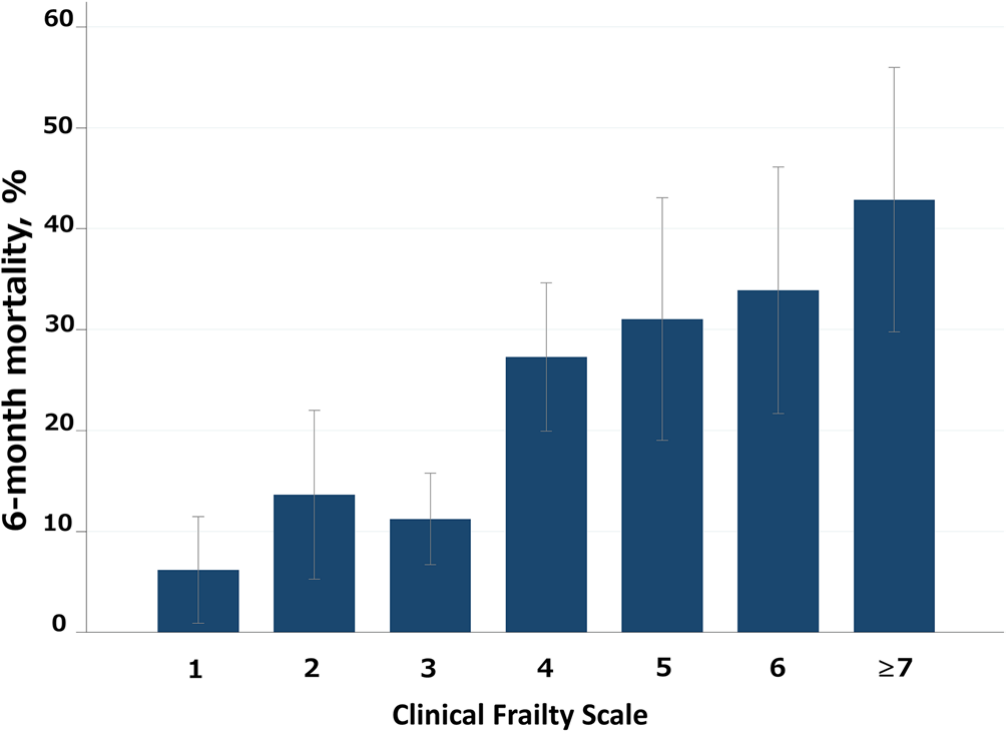
Six-month mortality by Clinical Frailty Scale score at the time of intensive care unit admission.

Six-month mortality increased as CFS scores increased (Fig. 2). Overall mortality at 6 months was 21% and ranged from 6.2% mortality in CFS 1 patients to 42.9% in CFS ≥ 7 patients. When adjusted for age, sex, CCI score, and APACHE II score, the RRs of one point increase of CFS was 1.19 (1.09–1.30). The crude and adjusted RRs for CFS 4 were 4.42 and 3.02, respectively (CFS 1 as a reference). The crude and adjusted RRs for CFS ≥ 7 were 6.94 and 3.60, respectively (Table 2). Similar results were obtained in sensitivity analyses that included only patients with complete case data (without imputation) (Supplementary eTable 5 online).

**Table 2:** Risk of mortality by CFS score in older patients with emergency ICU admission.

	Six-month mortality, n/N (%)	Risk ratio (95% CI) for mortality	
		Crude	Adjusted^a^
Per one point increase in CFS score		1.34 (1.24–1.45)	1.19 (1.09–1.30)
CFS score			
1	5/81 (6.2)	Reference	Reference
2	9/66 (13.6)	2.21 (0.78–6.28)	2.11 (0.76–5.93)
3	21/187 (11.3)	1.82 (0.71–4.66)	1.52 (0.61–3.79)
4	39/143 (27.3)	4.42 (1.81–10.77)	3.02 (1.24–7.32)
5	18/58 (31.0)	5.03 (1.98–12.78)	2.78 (1.08–7.20)
6	20/59 (33.9)	5.49 (2.19–13.8)	3.23 (1.24–8.39)
≥ 7	24/56 (42.9)	6.94 (2.82–17.11)	3.60 (1.42–9.11)

*CFS* Clinical Frailty Scale, *CI* confidence interval.

^a^Adjusted for age, sex, Charlson Comorbidity Index score, and APACHE II score.

### Stratified analysis of the primary outcome

When the patients were stratified by several potentially relevant clinical factors, frailty had a significant impact on mortality in patients 85 years of age or older and in patients between 65 and 85 years of age (Supplementary eFigure 1 online). Similarly, frailty increased the risk of mortality whether or not mechanical ventilation was required during the ICU stay and in patients with different illness severity, as indicated by APACHE II score (≥ 23 or < 23) (Supplementary eFigure 1 online).

### Secondary outcomes

**Figure 3:**
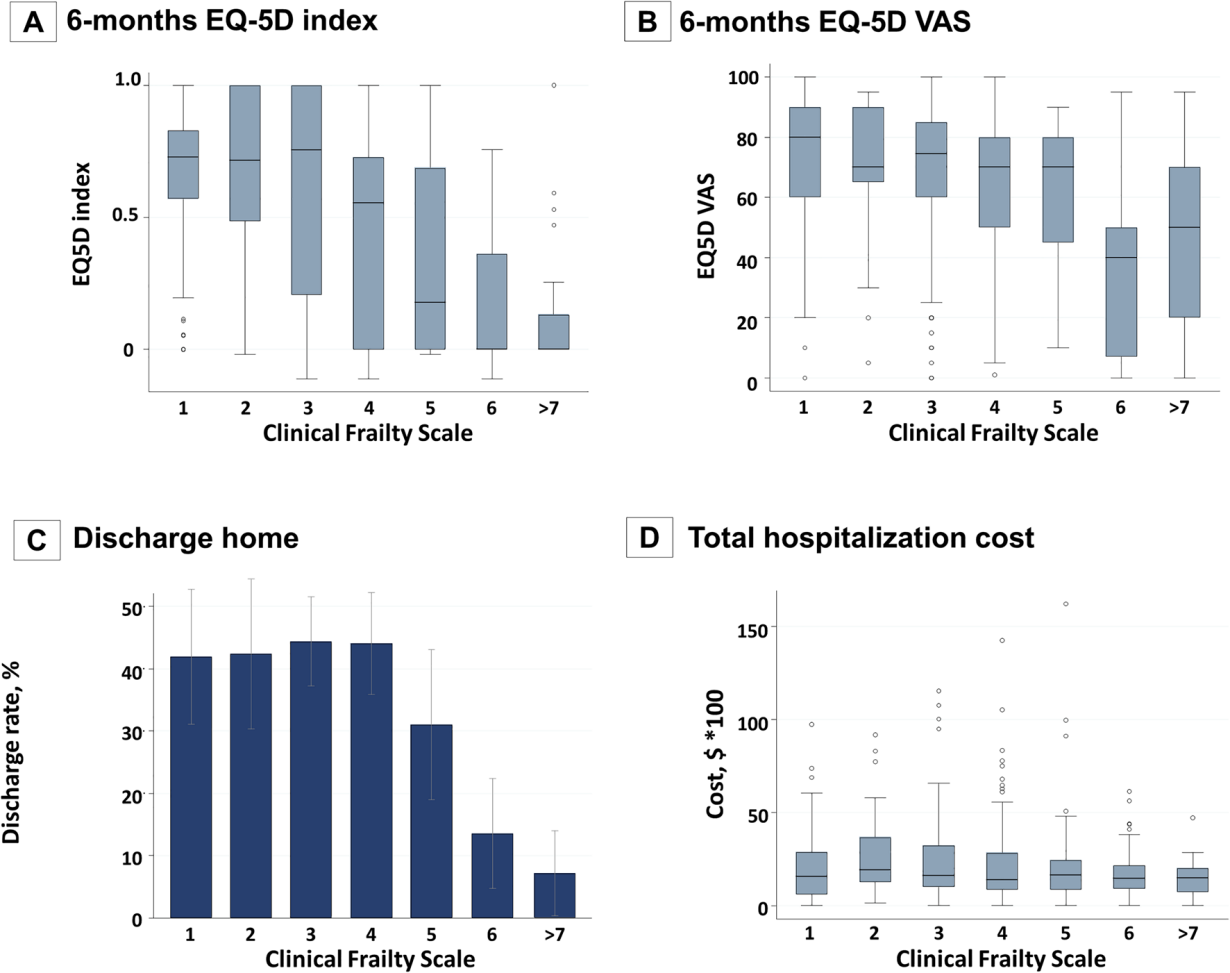
Association between each Clinical Frailty Scale score and secondary outcomes. (**A**) Quality of life as indicated by the EuroQol 5 Dimensions (EQ5D) index. (**B**) Quality of life as indicated by the EQ-5D using a Visual Analog Scale (VAS). (**C**) Discharge home. Home was defined as home of the patient or a relative (other than hospitals or nursing homes). (**D**) Total hospitalization cost. The actual cost of hospitalization on a piece-rate basis. Converted from Yen to US Dollars.

Figure 3 shows the association between secondary outcomes and CFS score. QOL (EQ-5D index and Visual Analog Scale) worsened as CFS score increased (Fig. 3A,B). The proportion of patients who could be directly discharged to their home or to the home of a relative decreased as CFS score increased (Fig. 3C). Overall, there was no association between total hospitalization cost and frailty (Fig. 3D).

## Discussion

This multicenter, prospective study of 650 critically ill patients 65 years of age or older demonstrated that baseline CFS score is an independent prognostic factor for mortality within 6 months of ICU admission, even after adjusting for age, sex, illness severity, and comorbidities. Furthermore, QOL worsened as baseline CFS score increased. However, there was no association between total hospitalization cost and baseline CFS. Assessment of frailty by attending physicians or nurses using baseline CFS in older patients before the acute illness could predict their long-term outcomes after emergency intensive care.

Although previous studies have investigated the impact of frailty on patients who require intensive care, they have been limited by their retrospective design, younger participant ages, and short observation periods^16–20^. Additionally, most of the previous studies assessed all ICU patients and included patients with planned surgeries^17–19,21,22^. One study examined prognostic performance of frailty in the emergency setting, but even the patients with low severity were included^23^. Our study was limited to critically ill patients who were admitted through the emergency department, and therefore provides information specifically on mortality risk in older patients requiring emergency intensive care. Additionally, most of the previous multi-institutional studies were conducted in Europe^10,20,21^. This study was conducted in Japan where the proportion of the population aged 65 years or older is extremely high (28%) and should provide pragmatic information for other parts of the world^24^.

Assessing frailty using the CFS may provide useful information for decision-making during treatment of older adults requiring intensive care and could suggest appropriate plans for long-term care with consideration of QOL after an emergency illness. We found that the patients' QOL after ICU admission was markedly worse if they had a CFS score ≥ 5. Similarly, the proportion of patients who could be discharged to their home or a relative’s home directly from the hospital decreased dramatically when their CFS score was ≥ 6.

It was surprising that there were no significant differences in the total medical costs incurred by patients with different degrees of frailty. This may reflect the difficulty in making ethical decisions regarding the appropriateness of intensive care for these patients^2^. However, some limitations may have been placed on the treatment of severely frail patients while in the ICU. There remains debate on the optimal distribution of health care expenditures for limited resources; however, age alone may not be used to recommend withdrawal of treatment^25^. Multiple factors should be considered when making treatment decisions^20^; our study emphasizes the importance of evaluating frailty.

Two major definitions have been developed to assess frailty: the frailty phenotype (FP), also known as Fried's definition or the Cardiovascular Health Study (CHS) definition^4^, and the frailty index (FI)^5^. Several indexes for assessing frailty have been developed from these frameworks, and 29 indexes were reviewed in 2016^26^. Although the gold standard for frailty determination is a comprehensive geriatric assessment performed by a specialist in geriatric medicine^27^, we chose the CFS derived from the FI to assess frailty in this study. We chose the CFS because this scale was recommended at international conferences and used in many recent studies^7,28^. The CFS is reliable indicator of patient outcomes in the intensive care setting and is one of the easiest assessment tools to use in the emergency setting^29–31^. CFS alone can be used to assess short-term mortality risk in older patients without the need for multiple prognostic indexes^32^. From our study results, we believe that obtaining a CFS score as well as a variety of other prognostic factors in the emergency department can be an aid to determine the indication for ICU admission and predict the prognosis of frail older patients.

Our study findings of median ICU mortality of 8.2% and 6-month mortality of 21.0% are lower than previously reported mortality rates in older patients with very similar illness severity (ICU mortality of 17.3–22.1% and 6-month mortality of 33–36.7%)^10,18,20–22^. The mortality rates of 6.2%, 13.6%, and 11.3% for CFS 1, CFS 2, and CFS 3, respectively, are particularly remarkable, indicating the relevance of treatment efforts and costs for older patients up to CFS 3. Differences in the Japanese medical system, the Japanese national health insurance^33,34^ system that makes care less costly for patients, and physicians' attitudes regarding treating older patients aggressively in the ICU^35^ may have contributed to the lower mortality rates as compared with other studies.

### Limitations

This study has several limitations. Family members or other surrogates participated in interviews about the patient’s condition and daily life prior to emergency medical care, and this information may have under- or over-estimated frailty. Because we focused on a critically ill patient population, baseline CFS score had to be assessed with information provided by the patient's surrogates in most cases. The response rate for the 6-month survey was 89.2%, which is very high compared with those of other studies in older adults^36,37^. It is possible, however, that the study underestimated the impact of frailty, because getting replies for patients in worse clinical condition, who required hospital readmission, or who had died may have been more difficult. Fortunately, a sensitivity analysis comparing patients with and without 6-month surveys suggested that the impact to the study was minimal. To accurately obtain clinical data relevant to illness severity and treatments, we limited our cohort to patients admitted to an ICU. It is possible that patients with more advanced frailty did not meet the criteria for ICU admission. Moreover, patients with missing baseline CFS score or patients from whom we could not obtain consent (from either the patient or their surrogate) were not included in the study, which may have caused selection bias. Finally, this was a single-nation study and might not be generalizable to other countries with different health care systems. However, Japan currently has one of the largest populations over the age of 65 in the world^24^, and most developed counties may face similar ethical and economic concerns in the near future^25,38^.

## Conclusions

We found that frailty is an independent predictor of long-term prognosis in the emergency intensive care setting. CFS score is an important predictor of outcomes in critically ill older adults who require emergency hospital admission.

## Supplementary Information

Supplementary Information.

## Data Availability

The datasets used and/or analyzed during the current study are available from the corresponding author on reasonable request.
