# Supplementary material for: Impact of frailty on long-term mortality in older patients receiving intensive care via the emergency department
Source: Sci Rep. 2023 Apr 3;13:5433. doi: 10.1038/s41598-023-32519-2 (PMC10070345; doi:10.1038/s41598-023-32519-2)
Supplement: Supplementary file 1 — Supplementary Information. [file 41598_2023_32519_MOESM1_ESM.docx]

**Online Supplementary Material**

**Impact of frailty on long-term mortality in older patients receiving intensive care via the emergency department**

**Mototaka Inaba, Hiromichi Naito, Takashi Yorifuji, Chikaaki Nakamichi, Hiroki Maeyama, Hideki Ishikawa, Nobuaki Shime, Sadayori Uemori, Satoshi Ishihara, Makoto Takaoka, Tsuyoshi Ohtsuka, Masahiro Harada, Satoshi Nozaki, Keisuke Kohama, Ryota Sakurai, Shuho Sato, Shun Muramatsu, Kazunori Yamashita, Toshihiko Mayumi, Kaoruko Aita, Atsunori Nakao, for the LIFE Study Investigators**

**eTable 1. Participating facilities and ethics committee names**

**eTable 2. Characteristics of patients with and without six-month survey**

**eTable 3. Follow-up patient data**

**eTable 4: Treatment limitations by Clinical Frailty Scale score**

**eTable 5.** **Sensitivity analyses that included only patients with complete case data (n=504)**

**eFigure 1. Stratified analysis of the primary outcome**

**eTable 1. Participating facilities and ethics committee names**

| **Participating Facility** | **Beds** | **ICU Beds** | **ED Visits** |
| --- | --- | --- | --- |
| National Hospital Organization Yokohama Medical Center | 510 | 10 | 13464 |
| Saiseikai Senri Hospital | 300 | 8 | 7845 |
| Yodogawa Christian Hospital | 582 | 12 | 28729 |
| National Hospital Organization Nagasaki Medical Center | 639 | 4 | 14000 |
| Okayamasaiseikai General Hospital | 473 | 10 | 18308 |
| Tsuyama Chuo Hospital | 518 | 20 | 17088 |
| Teikyo University Hospital | 1078 | 30 | 13717 |
| Nagasaki University Hospital | 874 | 19 | 2277 |
| Hyogo College of Medicine | 963 | 18 | 846 |
| Hiroshima University Hospital | 726 | 20 | 3390 |
| Hyogo Emergency Medical Center | 30 | 12 | 1203 |
| National Hospital Organization Kumamoto Medical Center | 550 | 6 | 18139 |
| Steel Memorial Hirohata Hospital | 783 | 18 | 9900 |
| Faculty of Medicine, Saga University | 604 | 6 | 3806 |
| University of Occupational and Environmental Health Hospital | 678 | 10 | 11681 |
| Okayama University Hospital | 855 | 12 | 1472 |
| Japanese Red Cross Medical Center | 708 | 8 | 23055 |

ICU=Intensive care unit, ED=Emergency department

**eTable 2. Characteristics of patients with and without six-month survey**

| **Characteristic** | **Total**  **(n=729)** | **Six-Month Survey Available**  **(n=650)** | **Without Six-Month Survey**  **(n=79)** |
| --- | --- | --- | --- |
| Age, median (IQR), years | 78 (72-84) | 79 (72-85) | 77 (70-84) |
| Sex (male) | 420 (57.6) | 380 (58.5) | 40 (50.6) |
| CCI score, median (IQR) | 5 (4-6) | 5 (4-6) | 5 (3-7) |
| APACHE II score, median (IQR) | 22 (16-29) | 22 (16-29) | 23.5 (19-31) |
| SOFA score, median (IQR) | 6 (4-9) | 6 (4-9) | 8 (4-10) |
| SAPS2 score, median (IQR) | 45 (34-60) | 44 (34-60) | 47 (34-63) |
| Lactate, median (IQR) | 2.3 (1.4-4.55) | 2.3 (1.4-4.5) | 2.6 (1.4-4.6) |
| ICU admission category |  |  |  |
| Cardiology | 156 (21.4) | 145 (22.3) | 11 (13.9) |
| Pulmonary | 79 (10.8) | 71 (10.9) | 8 (10.1) |
| Gastrointestinal | 107 (14.7) | 93 (14.3) | 14 (17.7) |
| Neurology | 168 (23.0) | 151 (23.2) | 17 (21.5) |
| Trauma | 110 (15.1) | 96 (14.8) | 14 (17.7) |
| Endocrine | 38 (5.2) | 33 (5.1) | 5 (6.3) |
| Skin/tissue | 9 (1.2) | 7 (1.1) | 2 (2.5) |
| Urology | 8 (1.1) | 7 (1.1) | 1 (1.3) |
| Others | 54 (7.4) | 47 (7.2) | 7 (8.9) |
| Exacerbation of chronic diseases | 113 (15.5) | 90 (13.8) | 23 (29.1) |
| Sepsis |  |  |  |
| none | 625 (85.7) | 562 (86.5) | 63 (80.8) |
| Sepsis | 48 (6.6) | 42 (6.5) | 6 (7.7) |
| Septic shock | 55 (7.6) | 46 (7.1) | 9 (11.5) |
| Living situation |  |  |  |
| Home without assistance | 613 (84.1) | 548 (84.3) | 65 (82.3) |
| Home with assistance | 51 (7.0) | 44 (6.8) | 7 (8.9) |
| Nursing home | 48 (6.6) | 42 (6.5) | 6 (7.6) |
| Hospital | 17 (2.3) | 16 (2.5) | 1 (1.3) |
| Less than high school education | 241 (33.1) | 211 (32.5) | 30 (38.0) |
| Dementia | 199 (27.3) | 169 (26.0) | 30 (38.0) |
| CFS score^a^ |  |  |  |
| 1 | 86 (11.8) | 81 (12.5) | 5 (6.3) |
| 2 | 73 (10.0) | 66 (10.2) | 7 (8.9) |
| 3 | 206 (28.3) | 187 (28.8) | 19 (24.1) |
| 4 | 167 (22.9) | 143 (22.0) | 24 (30.4) |
| 5 | 64 (8.8) | 58 (8.9) | 6 (7.6) |
| 6 | 69 (9.5) | 59 (9.1) | 10 (12.7) |
| 7 | 49 (6.7) | 43 (6.6) | 6 (7.6) |
| 8 | 15 (2.1) | 13 (2.0) | 2 (2.5) |
| Barthel Index score, median (IQR) | 100 (85-100) | 100 (85-100) | 100 (75-100) |
| High income | 230 (31.6) | 205 (31.5) | 25 (31.6) |

APACHE II=Acute Physiology and Chronic Health Evaluation II; CCI=Charlson Comorbidity Index; CFS=Clinical Frailty Scale; ICU=intensive care unit; SAPS 2=Simplified Acute Physiology; SOFA=Sequential Organ Failure Assessment.

Barthel Index: Ordinal scale used to measure performance in activities of daily living

Data are presented as median (IQR) for continuous measures, and n (%) for categorical measures

^a^No patient was scored as CFS 9.

**eTable 3. Follow up patient data**

|  | **No (%)** | | |
| --- | --- | --- | --- |
|  | **Total**  **(n=650)** | **Survivors**  **(n=514)** | **Death within Six Months**  **(n=136)** |
| **Mechanical ventilation** |  |  |  |
| IPPV | 222 (34.2) | 152 (29.6) | 70 (51.5) |
| NPPV | 42 (6.5) | 31 (6.0) | 11 (8.1) |
| **Tracheostomy** | 42 (6.5) | 29 (5.6) | 13 (9.6) |
| **ECMO** | 13 (2.0) | 9 (1.8) | 4 (2.9) |
| **Renal replacement therapy** | 59 (9.1) | 36 (7.0) | 23 (16.9) |
| **Limitation of medical treatments** | 121 (18.6) | 42 (8.2) | 79 (58.1) |
| DNAR | 104 (16.0) | 36 (7.0) | 68 (50.0) |
| withhold / withdraw | 41 (6.3) | 12 (2.3) | 29 (21.3) |
| **ICU length of stay, days** | 3.4 (1.6-7.7) | 3.1 (1.5-6.9) | 4.9 (1.7-10.7) |
| **Hospital length of stay, days** | 16.1 (7.8-29.0) | 17.6 (9.3-29.6) | 10.9 (3.9-26.6) |
| **Place of discharge^a^** |  |  |  |
| Other hospital | 314 (48.5) | 267 (52.1) | 47 (34.6) |
| Nursing home | 14 (2.2) | 14 (2.7) | 0 (0.0) |
| Home | 238 (36.7) | 231 (45.1) | 7 (5.1) |
| **Treatment costs, median (IQR), US dollars** | $15,655  (8,942-27,520) | $15,850  (9,085-27,324) | $14,407  (7,475-28,181) |

DNAR=do not attempt resuscitation order; ECMO=extracorporeal membrane oxygenation; IPPV=invasive positive-pressure ventilation; IQR=interquartile range; ICU=intensive care unit; NPPV=noninvasive positive-pressure ventilation.

Data are presented as median (IQR) for continuous measures, and n (%) for categorical measures.
Missing data: ICU length of stay, one patient; Hospital length of stay, two patients; Place of discharge, two patients.

^a^82 patients died in the hospital and were not included in the number of discharges

**eTable 4: Treatment limitations by Clinical Frailty Scale (CFS) score**

|  | **Total**  **(n=649)** | **CFS 1**  **(n-81)** | **CFS 2**  **(n=66)** | **CSF 3**  **(n=186)** | **CFS 4**  **(n=143)** | **CFS 5**  **(n=58)** | **CFS 6**  **(n=59)** | **CFS 7**  **(n=43)** | **CFS 8**  **(n=13)** |
| --- | --- | --- | --- | --- | --- | --- | --- | --- | --- |
| Any limitation of  medical treatments | 121 (18.6) | 3 (3.7) | 6 (9.1) | 19 (10.2) | 32 (22.4) | 15 (25.9) | 23 (39.0) | 16 (37.2) | 7 (53.8) |
| DNAR | 104 (16.0) | 2 (2.5) | 6 (9.1) | 16 (8.6) | 29 (20.3) | 12 (20.7) | 18 (30.5) | 15 (34.9) | 6 (46.2) |
| Withhold / Withdraw | 41 (6.3) | 1 (1.2) | 1 (1.5) | 8 (4.3) | 9 (6.3) | 7 (12.1) | 9 (15.3) | 4 (9.3) | 2 (15.4) |

Data are presented as n (%). DNAR=do not attempt resuscitation order.

**eTable 5.** **Sensitivity analyses that included only patients with complete case data (n=504)**

|  | **Six-Month Mortality**  **n/N (%)** | **Risk Ratio (95% CI)** | |
| --- | --- | --- | --- |
|  |  | **Crude** | **Adjusted^a^** |
| **One point increase in CFS score** |  | 1.32 (1.22 to 1.43) | 1.17 (1.07 to 1.29) |
|  |  |  |  |
| **CFS score** |  |  |  |
| 1 | 5/65 (7.7) | Reference | Reference |
| 2 | 8/50 (16.0) | 2.08 (0.72 to 5.98) | 1.99 (0.71 to 5.59) |
| 3 | 17/140 (12.1) | 1.58 (0.61 to 4.10) | 1.28 (0.52 to 3.19) |
| 4 | 36/114 (31.6) | 4.11 (1.69 to 9.95) | 2.65 (1.11 to 6.35) |
| 5 | 18/47 (38.3) | 4.98 (1.99 to 12.47) | 2.58 (1.02 to 6.52) |
| 6 | 14/42 (33.3) | 4.33 (1.68 to 11.15) | 2.54 (0.96 to 6.71) |
| >7 | 22/46 (47.8) | 6.22 (2.54 to 15.22) | 3.16 (1.26 to 7.94) |

CFS=Clinical Frailty Scale; CI=confidence interval

^a^Adjusted for age, sex, CCI score, and APACHE II score

**
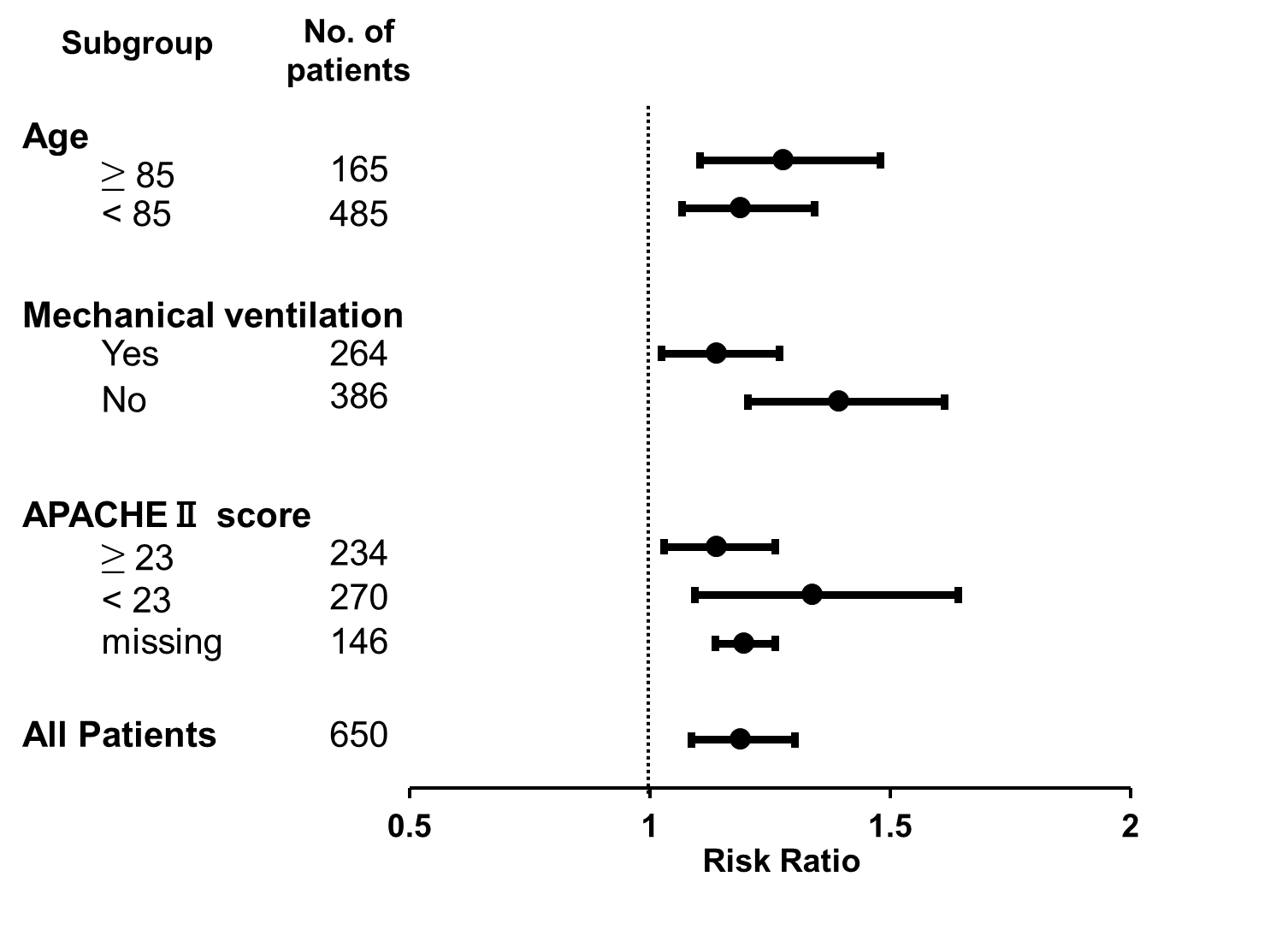
 eFigure 2. Stratified analysis of the primary outcome.**

The stratified analyses were conducted by mutually adjusting for other confounders in each stratum (e.g., age, sex, and CCI for APACHE II category). APACHE II score was imputed using multiple imputation in the adjustment for age and mechanical ventilation categories as well as for all patients. Error bars indicate 95% confidence intervals.
